# Muscarinic receptor agonists in animal models of psychosis: protocol for a systematic review and meta-analysis

**DOI:** 10.12688/f1000research.155356.1

**Published:** 2024-09-06

**Authors:** Spyridon Siafis, Nobuyuki Nomura, Johannes Schneider-Thoma, Irene Bighelli, Alexandra Bannach-Brown, Fiona J. Ramage, Francesca Tinsdeall, Ioannis Mantas, Sameer Jauhar, Sridhar Natesan, Anthony C. Vernon, Andrea de Bartolomeis, Sabine M. Hölter, Natascha I. Drude, Ulf Tölch, Wulf-Peter Hansen, Virginia Chiocchia, Oliver D. Howes, Josef Priller, Malcolm R. Macleod, Georgia Salanti, Stefan Leucht

**Affiliations:** 1 Technical University of Munich, School of Medicine and Health, Department of Psychiatry and Psychotherapy, Munich, Germany; 2 German Center for Mental Health (DZPG), partner site München/Augsburg, Munich, Germany; 3 Department of Neuropsychiatry, Keio University School of Medicine, Tokyo, Japan; 4 QUEST Center for Responsible Research, Berlin Institute of Health at Charité - Universitätsmedizin Berlin, Berlin, Germany; 5 Centre for Clinical Brain Sciences, The University of Edinburgh, Edinburgh, UK; 6 Department of Clinical Neuroscience, Karolinska Institutet, Stockholm, Sweden; 7 Department of Psychological Medicine, Institute of Psychiatry, Psychology and Neuroscience (IoPPN), King’s College London, London, UK; 8 Department of Psychosis Studies, Institute of Psychiatry, Psychology and Neuroscience (IoPPN), King’s College London, London, UK; 9 Psychiatric Imaging Group, MRC London Institute of Medical Sciences, Hammersmith Hospital, Imperial College London, London, UK; 10 Department of Basic and Clinical Neuroscience, School of Neuroscience, Institute of Psychiatry, Psychology and Neuroscience (IoPPN), King’s College London, London, UK; 11 MRC Centre for Neurodevelopmental Disorders, King's College London, London, UK; 12 Section of Psychiatry, Laboratory of Translational and Molecular Psychiatry and Unit of Treatment-Resistant Psychosis, Department of Neuroscience, Reproductive Sciences and Dentistry, University Medical School of Naples "Federico II", Naples, Italy; 13 Institute of Developmental Genetics, Helmholtz Center Munich, German Research Center for Environmental Health, Neuherberg, Germany; 14 BASTA-Bündnis für psychisch erkrankte Menschen, Munich, Germany; 15 Institute of Social and Preventive Medicine (ISPM), University of Bern, Bern, Switzerland; 16 Neuropsychiatry and Laboratory of Molecular Psychiatry, Charité - Universitätsmedizin Berlin and DZNE, Berlin, Germany; 17 University of Edinburgh and UK DRI, Edinburgh, UK

**Keywords:** antipsychotic; schizophrenia; psychosis; muscarinic receptor; acetylcholine; meta-analysis

## Abstract

**Background:**

Muscarinic receptor agonism is a promising mechanism of action for treating psychosis, not present in most D _2_R-blocking antipsychotics. Xanomeline, an M1/M4-preferring agonist, has shown efficacy in late-stage clinical trials, with more compounds being investigated. Therefore, we aim to synthesize evidence on the preclinical efficacy of muscarinic receptor agonists in animal models of psychosis to provide unique insights and evidence-based information to guide drug development.

**Methods:**

We plan a systematic review and meta-analysis of *in vivo* animal studies comparing muscarinic receptor agonists or positive allosteric modulators with control conditions and existing D2R-blocking antipsychotics in animals subjected to any method that induces behavioural changes of relevance for psychosis. We will identify eligible studies by searching multiple electronic databases. At least two independent reviewers will conduct the study selection and data extraction using prespecified forms and assess the risk of bias with the SYRCLE’s tool. Our primary outcomes include locomotor activity and prepulse inhibition measured with standardized mean differences. We will examine other behavioural readouts of relevance for psychosis as secondary outcomes, such as social interaction and cognitive function. We will synthesize the data using multi-level meta-analysis with a predefined random-effects structure, considering the non-independence of the data. In meta-regressions we will explore potential sources of heterogeneity from a predefined list of characteristics of the animal population, model, and intervention. We will assess the confidence in the evidence considering a self-developed instrument thatconsiders the internal and external validity of the evidence.

**Protocol registration:**

PROSPERO-ID: CRD42024520914

## Introduction

Antipsychotic drugs that block the dopamine 2 receptor (D2R) have been the cornerstone of pharmacological treatment for schizophrenia for over 70 years. ^1^^–^^3^ These drugs have demonstrated efficacy in reducing symptoms of psychosis, particularly positive symptoms such as hallucinations and delusions, ^1^ yet approximately one-third of patients exhibit an inadequate response to these treatments. ^4^ This mechanism of action also targets a downstream pathway of the aetiopathophysiology of psychosis, with limited efficacy on other core domains, such as negative symptoms like blunted affect and social withdrawal, as well as cognitive impairment. ^5^ Moreover, the risk-to-benefit ratio of antipsychotics is often challenged by their multiple side-effects. ^1^^,^^2^ Therefore, there has been a recognized need for more efficacious and tolerable medications for treating psychosis, but previous attempts to develop non-dopaminergic drugs have long been unsuccessful. ^3^^,^^6^

Muscarinic acetylcholine receptor agonism has recently been recognized as promising mechanism of action in the treatment of psychosis that can target components of the pathophysiology underlying schizophrenia, a property not present in most D2R-blocking antipsychotics. ^7^^–^^9^ Xanomeline, a muscarinic M1/M4-preferring receptor agonist, has demonstrated improvements in symptoms of schizophrenia in early and late-stage randomized placebo-controlled controlled trials with medium-to-large effect sizes, with potential cholinergic adverse events mitigated by its combination with trospium, a peripheral muscarinic antagonist. ^10^^–^^13^ Emraclidine, a drug with a different mode of action, acting as a selective M4 positive allosteric modulator (PAM), has also shown promising findings in an early clinical trial, ^14^ and additional muscarinic agents (e.g., NBI-1117568) ^15^ are under development.

Several unanswered questions remain however, including the comparative efficacy of different muscarinic receptor agonists, a comparison with existing D2R-blocking antipsychotics, the potential clinical differences between orthosteric agonists and PAMs, the specific contribution of individual muscarinic receptor subtypes (M1-M5), and their potential effects on specific symptom domains. In this context, preclinical studies can provide early insights and inform further drug development. Their large number and limitations in terms of internal and external validity, however, make the translation of their findings challenging. For this reason, a critical synthesis of their evidence is required, but none exists.

### Objectives

We therefore plan a systematic review and meta-analysis on the effects of muscarinic receptor agonists in animal models of relevance for psychosis concerning behavioural and motor outcomes as compared to control conditions and existing D2R-blocking antipsychotics. We will also carefully assess the potential biases of these studies and evaluate the confidence in the evidence, ultimately providing evidence-based information to facilitate future drug development in schizophrenia.

## Protocol

The protocol of the review is reported according to the PRISMA statement for protocols (PRISMA-P) ^16^ (see the checklist in the extended data), the guidelines from SYRCLE ^17^^,^^18^ and CAMARADES. ^19^ The protocol was registered with PROSPERO (ID: CRD42024520914) on 04.04.2024. The methodology of the protocol has been informed by our previous systematic review and meta-analysis conducted with the GALENOS project, ^20^ which examined trace-amine associated receptor 1 (TAAR1) agonists in animal models of psychosis. ^21^^,^^22^

### Study eligibility criteria and outcomes

*Study design*

We will include *in vivo* animal experimental studies examining any muscarinic receptor agonist versus inactive or active comparison groups in animal models of relevance for psychosis, as detailed below. There will be no restrictions on the inclusion criteria in terms of the randomization, blinding or other factors related to risk of bias, unit of allocation, duration of the study, publication status, year, country and language. We will exclude uncontrolled preclinical experiments, observational studies, and literature reviews.

*Animal population and model induction*

We will include animals that have undergone laboratory methods to induce psychosis-like behaviours and features. There are numerous models fitting this description, each with varying degrees of validity and unique strengths and weaknesses, but none are considered the gold standard. ^23^^–^^30^ Therefore, we will include the “classical” pharmacological models of psychosis and their behavioural readouts (see “Outcomes”), which have been widely utilized in drug discovery and possess some predictive validity, especially for positive symptoms, including the administration of psychostimulants (e.g., amphetamine, cocaine) or N-methyl-D-aspartate (NMDA) receptor antagonists (e.g., phencyclidine, ketamine, MK-801). ^29^^,^^31^^,^^32^ Other methods of induction will also be eligible and can include other pharmacological models, neurodevelopmental methods, lesion methods, genetic models, and combinations of different methods. ^24^^–^^30^ Such relatively broad inclusion criteria were utilized in our previous systematic review, ^21^^,^^22^ and any decision on the eligibility of the psychosis models will be made in consultation with experts in preclinical research (MRM, AB, FJ, FT, IM, SN, AdB, SH, NID, UT).

There will be no restriction on species, strain, age, and sex. Regarding genetic composition, we will include both wildtype animals and those that have undergone genetic interventions, if these interventions belong to eligible methods of induction (see above). Moreover, in the eligible studies, we will extract data from “naïve” animal cohorts (i.e., animals that have not undergone models of psychosis), and animal cohorts that have undergone both models of psychosis and muscarinic receptor antagonism via genetic or pharmacological manipulation. The eligible animal cohorts can be found in Table 1.

**Table 1.** Eligible animal cohorts and planned comparisons.

Animal cohort	Interventions received by the animal cohort					Planned comparisons with the comparison groups
	Model for psychosis	Muscarinic receptor agonists	D2R-blocking antipsychotics	Vehicle only or no treatment	Muscarinic receptor antagonism	
Muscarinic receptor agonists	Yes	Yes	No	No	No	Inactive and active control conditions
Combination of muscarinic receptor agonists and D2R-blocking antipsychotics	Yes	Yes	Yes	No	No	Active control conditions
Inactive control condition	Yes	No	No	Yes	No	Not applicable (comparison group)
Active control condition	Yes	No	Yes	No	No	Not applicable (comparison group)
Muscarinic receptor agonists in the context of muscarinic antagonism	Yes	Yes	No	No	Yes	Inactive control conditions with or without muscarinic receptor antagonism (comparison group)
Sham procedures	No	No	No	Yes	No	Not applicable (it will be used for the calculation of normalized mean differences)

We will aim to extract data from additional animal cohorts, if available, and consider additional potential comparisons if sufficient data are available.

We will exclude animals that have undergone induction methods for other specific conditions (e.g., transgenic models of Alzheimer’s disease, ^33^ the valproic acid-induced model of relevance for autism ^34^ and methods aiming to specifically model depressive-like behaviours, such as animal models of physical, social, or chronic mild stress). ^35^ We will also exclude *in vitro*, *ex vivo*, *in silico* studies and studies in humans. We will however consider extracting data from *ex vivo* measurements (e.g., Fos expression, autoradiography) following eligible *in vivo* experiments, potentially analysed in secondary publications (see “Outcomes”).

### Interventions

We will include any pharmacological agent acting as an agonist or positive allosteric modulator at any of the five subtypes of muscarinic acetylcholine M1-M5 receptors. There will be no restrictions on their receptor selectivity, pharmacological potency and efficacy (e.g., full or partial agonists), dose, timing of administration relative to the induction method, pharmacokinetic properties, or route of administration, provided the method is suitable for achieving effects in the central nervous system. These pharmacological agents can be administered individually or in combination with D2R-blocking antipsychotics or other medications, and combinations can be considered as distinct interventions. We will also consider data on the effects of muscarinic receptor agonists in the context of muscarinic receptor antagonism (see “Animal Population and model induction”) to evaluate whether the effects depend on the activation of muscarinic receptors or other mechanisms ( Table 1).

We will exclude clozapine, an existing antipsychotic acting on multiple neurotransmitter receptor systems and also as muscarinic receptor partial agonist, ^2^^,^^8^ which will be considered as a control intervention (see “Comparison groups”). Clozapine’s metabolite N-desmethylclozapine however, which has low affinity for dopamine receptors and acts as a muscarinic M1/M4 receptor agonist, ^36^ will be considered among the experimental interventions. Additionally, we will exclude pharmacological agents with different mechanisms of action (e.g., nicotinic acetylcholine receptor agonists, acetylcholinesterase inhibitors) and non-pharmacological interventions, including genetic interventions for muscarinic receptor overexpression.

*Comparison groups*

We will include the following comparison groups: i) inactive control conditions, consisting of animals undergoing models of a method to induce psychosis-like behaviour and features and receiving vehicle (e.g., injection of saline) or no treatment, and ii) active control conditions, consisting of the aforementioned animal cohorts treated with D2R-blocking antipsychotics. D2R-blocking antipsychotics will be defined as those medications listed in the Anatomical Therapeutic Chemical (ATC) classification with a code of N05A, except for lithium (N05AN01, a mood stabilizer primarily used for the treatment of bipolar disorder), pimavanserin (N05AX17, a 5-HT2A antagonist indicated for Parkinson’s disease psychosis but not schizophrenia), and any muscarinic receptor agonists approved by any regulatory body for schizophrenia (which will be considered as experimental interventions in this review), with no further restrictions.

We will also consider data in the eligible studies from sham procedures, consisting of “naïve” animal cohorts not subjected to methods of inducing psychosis-like behaviours or features and receiving either vehicle or no treatment, to calculate normalized mean differences in a sensitivity analysis (see “Effect sizes”).

### Outcome measures

There is no gold standard measure of preclinical antipsychotic efficacy due to the limited homology with the clinical symptoms of psychosis and the lack of an established biomarker. ^6^^,^^25^^,^^26^^,^^29^^–^^32^^,^^37^^–^^39^ Therefore, we aim to provide a comprehensive assessment of the effects of muscarinic receptor agonists by using data from a broad range of behavioral measures in various animal models of relevance for psychosis (see “Animal population and model induction”). This approach will facilitate the identification of strong clinical canditates. ^24^^,^^25^^,^^30^^–^^32^

We will examine as co-primary outcomes the effects on i) locomotor activity and ii) prepulse inhibition of the acoustic startle reflex, as these have been widely used with some predictive validity to identify antipsychotic effects in animal models of relevance for psychosis (see “Animal population and model induction”). ^21^^,^^26^^,^^31^ Furthermore, we will examine additional behavioral measures as secondary outcomes, including potential proxies for positive symptoms (e.g., stereotypies, hallucinatory-like percepts), ^40^ negative symptoms (e.g., lack of social interaction, operant-based motivational tasks), ^31^^,^^41^ depressive- (e.g., forced swim, tail suspension, sucrose preference tasks) ^31^^,^^41^ and anxiety-like behaviors (e.g., elevated plus maze), and cognitive function (e.g., tests recommended by the CNTRICS initiative). ^30^^,^^37^^,^^39^ The eligibility and any potential grouping of the behavioral measures will be evaluated in collaboration with experts in preclinical research prior to commencing data analysis, and any decisions will be documented.

We will exclude non-behavioural outcomes, such as histopathological and neurobiological measures due to the lack of an established biomarker, ^6^ and adverse events due to the inconsistent and scarce reporting in animal studies, as identified in our previous review. ^21^ Nonetheless, we will aim to extract their data, if available, and potentially analyse them in secondary publications.

### Study identification

We will conduct searches in PubMed, MEDLINE via Ovid, Web of Science, EMBASE, PsychINFO, from database inception using keywords for psychosis, muscarinic agents, and animal filters, ^42^ similar to a previous review. ^21^^,^^22^ For muscarinic agents, we will use broad terms like “muscarinic agonists” and the names/synonyms of specific relevant agents identified from IUPHAR/BPS ^43^ and previous reviews. ^7^^,^^44^ There will be no restrictions such as on language or publication date. The search strategies will be developed in collaboration with information specialist (see “Acknowledgment”). A draft search strategy in MEDLINE via Ovid can be found in the extended data, and the final search strategies will be reported according to the PRISMA statement for reporting literature searches (PRISMA-S). ^45^

We plan additional searches to improve the coverage of the study identification and identify potentially unpublished studies:

1) We will aim to search in the Systematic Online Living Evidence Summaries for preclinical psychosis research ( psychosis-SOLES), ^46^ which is a dedicated and continuously updated database utilizing machine learning and text mining algorithms.
2) We will search preclinical animal study registries (i.e., animalstudyregistry.org, https://preclinicaltrials.eu/), although pre-registration of animal studies has not been widely adopted.
3) We will aim to search preprint registries (i.e., medRxiv, bioRxiv), Google patents, specific journals of neuropsychopharmacology.
4) We will inspect reference lists of included studies, previous reviews, ^7^^,^^8^^,^^44^^,^^47^ and conference proceedings published within the last 20 years.
5) We will contact the first/corresponding author of included studies and pharmaceutical industries of muscarinic agents for additional studies and/or missing data in their studies. We will send emails with two follow-up reminders in case of no response.

### Study selection

At least two independent reviewers will screen in the Systematic Review Facility (SyRF) ^23^ the de-duplicated records identified in the searches in two phases: i) title and abstract, and ii) full-text screening. Any discrepancy between the two reviewers will be resolved through discussion with a more senior reviewer. If not resolved by discussion, the full-text of the study will be acquired (if at the title/abstract level) or additional information from the original study authors will be obtained (if at the full-text level).

At the title/abstract screening, records will be excluded according to the following hierarchy: i) review articles, ii) not referring to *in vivo* animal study, iii) not referring to muscarinic receptors or agents acting on them.

At the full-text screening, records will be excluded from the review and/or meta-analysis according to the following hierarchy against the eligibility criteria: i) ineligible study design, ii) ineligible animals/population, iii) ineligible intervention, iv) ineligible comparison groups, v) ineligible outcome measure, vi) inadequate reporting of outcome data. If possible, we will consider the studies excluded in terms of their reported outcomes in the assessment of reporting bias (see “Data synthesis”). The study selection process and the reasons for exclusions at the full-text level will be reported in a flow diagram. ^48^

### Data extraction

At least two independent reviewers will conduct the data extraction in SyRF ^23^ using pre-specified data extraction forms that will be adapted from a previous systematic review. ^21^ Discrepancies between the two reviewers will be reconciled by a more senior reviewer, or if not possible, by contacting study authors for additional information.

We will extract data regarding study identification (e.g., author names, title, publication year), study design (e.g., risk of bias, reporting completeness), animal population and model induction (e.g., age, sex, species strain, body weight, characteristics of the induction method), experimental and control interventions (e.g., type, dose and timing of the administration of drugs), and outcome measures (e.g., exact name of the behavioural task, methods of measurement, quantitative data). We will seek information from various sources in the following order of priority: i) text and tables, ii) figures using WebPlotDigitizer version 4 (a free and opensource tool distributed under GNU Affero General Public License Version 3), ^49^ iii) contacting authors for missing information, and iv) employing imputation methods.

We will extract quantitative data for the outcomes anticipated to be reported as continuous measures. Endpoint and change scores will both be eligible and jointly synthesized. ^50^ When both are reported, we will prefer endpoint scores because they do not require baseline assessments and are expected to be the most frequently reported in the eligible animal experiments. We will extract the unit of measurement, mean, standard deviation and the number of participants that these correspond to. We will apply a minus transformation whenever appropriate to harmonize the direction of effects across the extracted data (e.g., a higher score indicating a better outcome). Missing standard deviations will be derived by the following methods according to their order of priority: i) calculation from standard error, ii) estimation from test statistics (e.g., p-values, t-tests, median and ranges), iii) contacting the authors of the original studies for additional information, iv) imputed from the standard deviations of other studies (although this method has not been validated in animal studies that often have small sample sizes). ^51^^,^^52^ If the exact number of animals is not reported, ^53^ we will estimate it with available information (e.g., using the lower boundary of a range, if reported) or consider imputation methods. If dichotomous measures are reported, we will extract the number of animals with the event and the total number of animals analysed. We will exclude studies with imputed data in a sensitivity analysis (see “Sensitivity analysis”).

For crossover trials, we will prefer data from the first phase to avoid carryover effects, but we will also use data from the entire phase by applying appropriate corrections considering the within-subject correlation. ^54^ These studies will be excluded in a sensitivity analysis (see “Sensitivity analysis”).

We will use data from any reported time point, but preference will be given for the longest time point following multiple administrations of the intervention over an extended period. If outcomes are measured multiple times after a single administration, we will consider calculating the area under the curve. ^22^^,^^55^

If multiple variations of the same outcome are reported, we will extract and jointly analyse them (see “Data synthesis”).

### Assessment of risk of bias and reporting completeness

Two independent reviewers will assess risk of bias using the SYRCLE’s tool considering domains for selection, performance, detection, attrition, reporting and other biases. ^56^ We will assign an overall high risk of bias to a study if at least one domain in the SYRCLE’s tool is assessed as having a high risk of bias. Since high-quality reporting is essential for assessing the risk of bias, two independent reviewers will also evaluate the completeness of reporting using a modified version of the ARRIVE Essential 10 checklist. ^22^^,^^57^ This is necessary because the reporting completeness of animal studies is often poor, frequently leading to unclear assessments of risk of bias. Any discrepancies between the two reviewers will be resolved through discussion with a more senior reviewer or by contacting the study authors for additional information.

### Assessment of indirectness of animal experiments

We expect that the findings from various animal models of relevance for psychosis will have different degrees of applicability to clinical trial settings. However, there is no established method for assessing their indirectness in the context of a systematic review. Moreover, there is limited synthesized evidence on the validity of animal models of relevance for psychosis, and we expect substantial variability in the methods of modelling and measuring psychosis-like behaviours in animals. ^25^^,^^26^ This makes it challenging to set predefined criteria for assessing the indirectness of animal experiments in the context of psychosis.

Nevertheless, we will aim to use the extracted data to provide an experiment-level judgment of indirectness as “low risk”, “high risk”, or “some concerns”, considering how closely the experiment reflects the clinical trial setting in terms of animal population, model induction, intervention, and outcome. This assessment will inform the evaluation of indirectness domain in the confidence in the evidence (see “Confidence in the evidence”).

To achieve this, we will evaluate the validity in animal experiments and the applicability of the intervention (e.g., treatment over an extended period, initiation of treatment after model induction) based on previous frameworks and checklists. ^21^^,^^58^^–^^62^ Specifically, we will apply the framework of Belzung and Lemoine ^62^ to assess the following domains (and sub-domains) of validity in animal experiments, i.e., homological (species strain), pathogenic (ontopathogenic, triggering), mechanistic, face (ethological, biomarker) and predictive (induction, remission). This framework can offer a more refined and systematic approach compared to the traditional domains of construct, face and predictive validity, which have often been inconsistently applied in the literature. ^25^^,^^62^^,^^63^ However, the exact methods will be determined *a posteriori* in consultation with experts in preclinical research.

### Data synthesis

*Planned comparisons*

Our main aim is to synthesize data for each outcome and for the comparisons described in Table-1. Meta-analysis will be conducted when there are at least two independent effect sizes for the same outcome, as in our previous systematic review. ^21^

We will examine the data and if there is reasonable consistency across the comparisons, we will consider network meta-analysis to examining the comparative effects of the different muscarinic receptor agonists, various D2R-blocking antipsychotics, and inactive control conditions. ^64^

*Effect sizes*

The main effect size will be the standardized mean difference (SMD) due to the varying measures and units of the behavioural outcome measures across the studies. We will also use normalized mean difference (NMD) in a sensitivity analysis. ^53^^,^^65^ If outcomes are reported as dichotomous, we will calculate odds ratios (ORs) and convert them into SMDs using the Hasselblad and Hedges method ^66^ to enable their combination with results of continuous measures.

In addition to the estimation of the average treatment effects, we also aim to conduct a meta-analysis of variation to estimate the inter-individual variability of the effects using the variability ratio (VR) or the coefficient of the variation ratio (CVR) in case a mean-variance relationship is expected. ^65^^,^^67^ This analysis will be conducted for the comparison of muscarinic receptor agonists to inactive control conditions.

*Data synthesis approach*

We will opt for synthesizing the data using multilevel meta-analytic models, which enable handling non-independent data. ^65^ We will use a predefined multilevel random-effects structure with nested levels, from higher to lower, for animal strain, study, and experiment, provided there are at least five distinct categories for at least one of the levels, as in our previous systematic review. ^21^ For non-independent sampling errors, we will estimate the within-study variance-covariance (VCV) matrix using any reported correlation in the original studies or assuming a correlation of 0.5 (see other assumed correlations in “Sensitivity analysis”). ^65^ The restricted maximum likelihood (REML) method will be used to estimate the between-study variance (τ ^2^) and between-study VCV. ^65^^,^^68^ We will adjust the confidence intervals using t- or F-distributions with degrees of freedom appropriate for the multilevel model. ^69^

To our knowledge, network meta-analysis has not been widely applied to the synthesis of animal experiments, and we anticipate several challenging issues, including the limited evidence for or against inconsistency, small sample sizes, and non-independent effect sizes. ^60^ We will examine whether the assumptions of a network meta-analysis can be fulfilled by comparing the distribution of potential effect modifiers across treatment comparisons and measuring incoherence using statistical tests. ^70^ Justifying these assumptions with preclinical data however, might be challenging. If a network meta-analysis is deemed feasible, we will extend the multilevel models and consider covariate-adjusted analysis (see “Exploration of heterogeneity”). The exact methodology will be defined *a posteriori*, and, if a network meta-analysis is justified, will be thoroughly reported in an amendment of the protocol before conducting it.

We will present the effect sizes with their 95% confidence intervals and prediction intervals.

*Exploration of heterogeneity*

We will quantify heterogeneity using the variance of the random effects with its components, and the 95% prediction intervals. We will explore potential sources of heterogeneity for each outcome through meta-regression or subgroup analyses, if sufficient data are available, considering the following tentative list of potential effect-modifiers: age, sex, species/strain, comorbidities, characteristics of the model of induction of psychosis-like behaviours (e.g., pharmacological or genetic, severity), muscarinic receptor agonists and their pharmacological characteristics (e.g., mode of action, selectivity for muscarinic or other receptors, potency, efficacy), dose, route of administration, duration of treatment, timing of the intervention (e.g., before or after model induction), co-treatments, characteristics of the study (e.g., publication year, reporting completeness, and risk of bias), and outcome measurements (e.g., different measures for locomotor activity). Additional variables will be considered if they are deemed relevant and there are sufficient data.

We will also aim to standardize doses across drugs to examine dose-effects relationships using the standardized dose as a covariate in a meta-regression. However, any potential standardization method cannot be predefined due to the pharmacological differences among various drugs ^21^ and differences across species/strains. ^71^

*Sensitivity analysis*

The robustness of the findings for each outcome will be examined through sensitivity analysis by: i) excluding studies with an overall high risk of bias (see “Assessment of risk of bias and reporting completeness”), ii) using normalized mean difference (see “Effect Sizes”), iii) excluding studies with imputed data (e.g., standard deviations, number of animals) and crossover studies reporting data from the entire period, iv) excluding interventions examined in single studies due to potentially inflated effect estimates, ^60^ v) assuming correlations of 0.2 and 0.8 in estimating the within-study VCV, ^65^ and vi) using a robust variance estimation to obtain cluster-robust standard errors. ^65^^,^^72^

*Reporting bias and small-study effects*

Publication and other non-reporting biases are highly prevalent in preclinical research, potentially having a substantial impact on the estimated efficacy. ^73^^,^^74^ We will aim to evaluate potential within- and across-study non-reporting biases by adapting the framework for assessing the risk of bias due to missing evidence (ROB-ME) in clinical trials, assigning ratings of “low risk”, “high risk”, or “some concerns”. ^75^^–^^77^ We will include both published and unpublished studies, although the latter may be difficult to find due to the limited adoption of pre-registration protocols. We will evaluate potential reasons of non-reporting of outcome data in studies excluded due to ineligible outcome measures or inadequate reporting of outcome data, although this may be more challenging due to the poor reporting quality of animal studies (see “Assessment of risk of bias and reporting completeness”). Moreover, we will explore small-study effects for each outcome using contour-enhanced funnel plots and multilevel regression-based tests, ^65^ using the square root of the sample size, when there are sufficient data from at least 10 studies.

*Confidence in the evidence*

We will evaluate the confidence in the evidence for each of outcome using a modified version of the GRADE framework ^58^^,^^59^ taking into account the domains of risk of bias (and reporting completeness), indirectness, heterogeneity, imprecision, and reporting bias, similar to our previous systematic review. ^21^ Ultimately, we will aim to draw an overall conclusion on the preclinical efficacy of muscarinic receptor agonists by considering the evidence from the different behavioural domains.

*Statistical software*

Data analysis will be conducted in R statistical software ^78^ using the package *metafor*, ^69^ along with other appropriate packages for data cleaning, specific meta-analytic models, and visualization. We will report the complete list of packages used along with their versions in the publication of the results.

### Dissemination of information

We plan to publish the systematic review and meta-analysis as open access in peer-reviewed journals, potentially resulting in multiple publications, and present the findings at conferences. Lay language summaries will be prepared and disseminated with the help of patient and relative groups, such as BASTA (Bündnis für psychisch erkrankte Menschen) and ApK (Aktionsgemeinschaft der Angehörigen psychisch Kranker e.V.). We will also make the methods, data, and code publicly available in a GitHub repository.

### Study status

As of the date of submission of this protocol on 18.08.2024, we have completed the preliminary searches and piloting of study selection process and started, but not completed, the full searches and the full screening of search results against the eligibility criteria. We have not yet started the data extraction, risk of bias and quality assessment, or data synthesis.

There were no changes from the original PROSPERO registration of the protocol, except for expanding the methods with additional details and deciding not to search Scopus and CINAHL after consulting with the information specialist (which is not expected to affect the coverage of our search). Any additional deviations or modifications will be reported along with the findings and in updates to the PROSPERO registration.

## Discussion

The planned systematic review and meta-analysis aims to evaluate the preclinical efficacy of muscarinic receptor agonists in animal models of relevance for psychosis. This will be achieved through a comprehensive search, advanced data synthesis methods, and a critical evaluation of potential risks of bias and confidence in the evidence. The systematic review has the potential to provide unique insights into important unanswered questions regarding muscarinic receptor agonism in the treatment of psychosis. It may also identify promising muscarinic agents or specific mechanisms of action, which could guide future drug development for schizophrenia.

Furthermore, conducting a systematic review to examine the preclinical efficacy of antipsychotics is a relatively novel approach. Only a few such reviews currently exist, ^21^^,^^26^^,^^79^ making this work particularly valuable. Therefore, our review has the potential to highlight the limitations of existing animal models of relevance for psychosis and address potential translational disconnects to improve the design of future preclinical research. ^6^^,^^25^^,^^31^

We focused on muscarinic receptor agonism due to its potential to target the underlying pathophysiology of schizophrenia and the promising findings for agonists of M1/M4 muscarinic receptors, ^7^^–^^9^ with xanomeline combined with trospium being reviewed for approval by the Food and Drug Administration. ^80^ Muscarinic receptor agonism has also the most advanced data compared to other novel mechanisms of action being investigated in the treatment of psychosis. ^9^ For example, there were encouraging results from a phase II trial for ulotaront, a dual TAAR1 agonist and 5-HT _1A_ partial agonist. ^81^ A recent synthesis of evidence from early- and late-stage clinical trials, and animal studies however, suggested that TAAR1 agonists may be less efficacious compared to existing D2R-blocking antipsychotics, but additional data are required to draw more definitive conclusions and more drugs are under development. ^21^

We anticipate limitations and challenges in conducting the review. There is no gold standard animal model for psychosis, ^6^^,^^25^^,^^31^ and cross-species differences in the muscarinic receptor system and the effects of their agonists may exist. ^8^ Therefore, we will include all relevant animal models and behavioural readouts of preclinical efficacy, critically evaluating the confidence in the evidence to provide a comprehensive synthesis with potential translational relevance. We will not analyse neurobiological measures due to their heterogeneous use across studies and the lack of an established biomarker in schizophrenia, ^6^ but they could further elucidate the mechanisms of muscarinic receptor agonism and its role in regulating dopaminergic signalling and other underlying pathophysiological mechanisms. ^8^^,^^47^ We will aim to extract and potentially analyse these measures in secondary publications. Additionally, the methods for systematic reviews of animal studies in psychiatry are not well established and may present unique challenges. ^82^ Our interdisciplinary team, which includes methodologists, statisticians, and clinical and preclinical researchers, will address any potential issues to ensure a robust synthesis of preclinical evidence.

In conclusion, our planned systematic review and meta-analysis will be the first to examine the preclinical efficacy of muscarinic receptor agonists for schizophrenia, with the potential to provide evidence-based information to guide future preclinical and clinical research on this topic.

## Ethics and consent

Not applicable.

## Authors contributions

Spyridon Siafis: Conceptualization, Methodology, Writing – Original Draft, Supervision, Project administration, Funding acquisition.

Nobuyuki Nomura: Writing – Review & Editing.

Johannes Schneider-Thoma: Methodology.

Irene Bighelli: Methodology.

Alexandra Bannach-Brown: Methodology, Writing – Review & Editing.

Fiona J. Ramage: Methodology, Writing – Review & Editing.

Francesca Tinsdeall: Methodology, Writing – Review & Editing.

Ioannis Mantas: Methodology, Writing – Review & Editing.

Sameer Jauhar: Methodology, Writing – Review & Editing.

Sridhar Natesan: Methodology, Writing – Review & Editing.

Anthony C. Vernon: Methodology, Writing – Review & Editing.

Andrea de Bartolomeis: Methodology, Writing – Review & Editing.

Sabine M. Hölter: Methodology, Writing – Review & Editing.

Natascha I. Drude: Methodology, Writing – Review & Editing.

Ulf Tölch: Methodology.

Wulf-Peter Hansen: Methodology, Writing – Review & Editing.

Virginia Chiocchia: Methodology, Writing – Review & Editing.

Oliver Howes: Methodology.

Josef Priller: Methodology.

Georgia Salanti: Methodology, Writing – Review & Editing.

Malcolm R. Macleod: Methodology, Writing – Review & Editing.

Stefan Leucht: Conceptualization, Writing – Original Draft, Writing – Review & Editing, Supervision, Project administration, Funding acquisition

## Data Availability

No data associated with this article. Zenodo: Protocol for meta-analysis on muscarinic receptor agonists in animal models of psychosis (ANIMUS-SR), https://doi.org/10.5281/zenodo.13378744. ^83^ This project contains the following extended data:
- Draft search strategy in MEDLINE via Ovid.pdf - PRISMA-P checklist.pdf Zenodo: PRISMA-P checklist for “Protocol for meta-analysis on muscarinic receptor agonists in animal models of psychosis (ANIMUS-SR)”, https://doi.org/10.5281/zenodo.13378744. ^83^ Data are available under the terms of the Creative Commons Attribution 4.0 International license (CC-BY 4.0).
