# Supplementary material for: Muscarinic receptor agonists and positive allosteric modulators in animal models of psychosis: protocol for a systematic review and meta-analysis
Source: F1000Res. 2025 Jan 2;13:1017. Originally published 2024 Sep 6. [Version 2] doi: 10.12688/f1000research.155356.2 (PMC11751611; doi:10.12688/f1000research.155356.2)
Supplement: Peer review history

# Reviewer report: Reviewer response for version 2

**DOI:** 10.5256/f1000research.176234.r357863

**Published:** 2025-01-21

**Authors:** Xiaonan Guo

**Affiliations:** 1 Zhejiang University, Hangzhou, Zhejiang, China

This paper is a comprehensive protocol studying muscarinic receptor agonists and positive allosteric modulators in animal models of psychosis. Generally, I think it will promote the clinical use and biological insight of the muscarinic receptor agonists and positive allosteric modulators in treating schizophrenia. The method is so perfect that no obvious limitations can be found. I only provide the minor suggestion to improve the article, which the authors could consider. 

 Since psychosis is strongly associated with dopamine function, it's better to introduce the influence of dopamine function when treating animal models of psychosis with muscarinic receptor agonists and positive allosteric modulators. Linking the muscarinic function with dopamine function will shed light on clinical translation of muscarinic agonist.

Is the study design appropriate for the research question?

Yes

Is the rationale for, and objectives of, the study clearly described?

Yes

Are sufficient details of the methods provided to allow replication by others?

Yes

Are the datasets clearly presented in a useable and accessible format?

Yes

Reviewer Expertise:

Brain, metabolic, and genetic mechanism in psychiatric disorders (especially in BD).

I confirm that I have read this submission and believe that I have an appropriate level of expertise to confirm that it is of an acceptable scientific standard.

# Reviewer report: Reviewer response for version 1

**DOI:** 10.5256/f1000research.170522.r325671

**Published:** 2024-10-24

**Authors:** William Messer

**Affiliations:** 1 Department of Medicinal and Biological Chemistry, College of Pharmacy and Pharmaceutical Sciences, Department of Pharmacology and Experimental Therapeutics,, University of Toledo, Toledo, OH, USA

The article outlines a protocol for a specific systematic review and meta-analysis examining the impact of muscarinic agonists (and allosteric modulators) in animal models of psychosis. As such, it is more a research proposal than a paper describing results.

 The authors indicate that D _2_ dopamine antagonists have been the cornerstone for schizophrenia therapy for the last 70 years, although atypical antipsychotics target other receptors, including 5-HT _2_ receptors. Such compounds, including clozapine and olanzapine, are linked to a different set of adverse effects than classical dopamine antagonists including hypoglycemia and weight gain. Nevertheless, the topic is timely given the recent approval of xanomeline, in combination with trospium, for the treatment of schizophrenia.

 A systematic review and a meta-analysis are proposed for examining the impact of muscarinic agonists and allosteric modulators in animal models of psychosis. The level of complexity is high with respect to the varying types of compounds (agonists vs. partial agonists, receptor subtype selectivity, binding affinity, etc.), which will make it difficult to accurately categorize compounds. For example, positive allosteric modulators often exhibit a range of effects that include modulation of acetylcholine potency and efficacy in addition to allosteric agonist activity. The degree to which such activities have been adequately assessed will likely vary from lab to lab and such data may not be readily available in the papers that address in vivo efficacy in animal models of psychosis. These concerns are addressed to some extent in the “Exploration of heterogeneity” section, but a more explicit discussion of how concepts of potency and efficacy, for example, will be handled is warranted. In addition to the sources of heterogeneity discussed, the physicochemical properties of compounds may impact the effectiveness of treatments; consideration of ADME properties of compounds could provide an important perspective. For example, multiparameter optimization values could help explain variability in responses for compounds with similar receptor activity profiles.

 Beyond the pharmacological properties of the compounds under investigation, the impact of muscarinic receptor activation might vary significantly depending on the different compounds utilized to induce behavioral changes (e.g., NMDA antagonists vs. dopamine agonists) or the behavioral readouts (e.g., pre-pulse inhibition of startle response vs. apomorphine induced climbing). The authors refer to some of the inherent difficulties in the “Assessment of indirectness of animal experiments” section, including concerns about the applicability of various animal models to clinical utility. In this sense, it might be helpful to examine the consistency of comparator compounds (e.g., D _2_ and 5-HT _2_ antagonists) that are used clinically in treatment of schizophrenia, and are often used as control compounds in studies of muscarinic agonists and other potential therapeutic compounds. This is perhaps alluded to in the “Data synthesis/planned comparisons” section, although additional details would be helpful.

 An additional consideration is the presence or absence of adverse effects for the compounds under investigation. The clinical utility of muscarinic agonists has been limited by adverse effect profiles, which vary from compound to compound, but likely include both off-target (e.g., M _3_ receptor-mediated) and on-target (i.e., M _1_ or M _4_ receptor-mediated) effects. Adverse effects such as salivation, lacrimation, diarrhea, or convulsions would provide important information regarding the *in vivo* pharmacological profile of compounds and the separation between beneficial and undesirable effects (i.e., the therapeutic window). 

 Overall, the topic is of timely interest and worth pursuing with some additional attention to the details to be evaluated.

Is the study design appropriate for the research question?

Yes

Is the rationale for, and objectives of, the study clearly described?

Yes

Are sufficient details of the methods provided to allow replication by others?

Partly

Are the datasets clearly presented in a useable and accessible format?

Not applicable

Reviewer Expertise:

My research focuses on the design and development of selective agonists and allosteric modulators of muscarinic receptors with potential utility in the treatment of neuropsychiatric disorders.

I confirm that I have read this submission and believe that I have an appropriate level of expertise to confirm that it is of an acceptable scientific standard, however I have significant reservations, as outlined above.

# Response

**Published:** 2024-12-20

**Authors:** Spyridon Siafis

**Affiliations:** Psychiatry and Psychotherapy, Technische Universitat Munchen, Munich, Bavaria, Germany

**#2.1: The article outlines a protocol for a specific systematic review and meta-analysis examining the impact of muscarinic agonists (and allosteric modulators) in animal models of psychosis. As such, it is more a research proposal than a paper describing results.**

Our reply:

 We would like to thank the reviewer for their constructive comments on our protocol, which have helped improve the clarity of the methods. This manuscript indeed corresponds to a protocol for a systematic review, and the results of the completed review will be published in a peer-reviewed journal, as outlined in the “Dissemination of information” section.

 Please find our point-by-point responses to the comments below.

**#2.2: The authors indicate that D _2_ dopamine antagonists have been the cornerstone for schizophrenia therapy for the last 70 years, although atypical antipsychotics target other receptors, including 5-HT _2_receptors. Such compounds, including clozapine and olanzapine, are linked to a different set of adverse effects than classical dopamine antagonists including hypoglycemia and weight gain. Nevertheless, the topic is timely given the recent approval of xanomeline, in combination with trospium, for the treatment of schizophrenia.**

Our reply:

 We agree with this point that although the primary mechanism of action for antipsychotics has historically been the blockade of dopamine D2 receptors, these drugs also interact with other neurotransmitter receptors, with adverse events often linked to their receptor-binding profiles. Accordingly, we have revised the introduction to incorporate this information and have also noted the recent approval of xanomeline-trospium, which had not been approved at the time of the initial manuscript submission.

Changes in the manuscript:

  

- We revised the introduction to mention the association of adverse events of antipsychotics with their receptor-binding profiles and off-target actions on other neurotransmitter receptors, as well as the approval of xanomeline-trospium for schizophrenia by the US FDA:

“Antipsychotic drugs that block the dopamine D2 receptor (D2R) have been the cornerstone of pharmacological treatment for schizophrenia for over 70 years. ^1^^ – ^^3^ …..Moreover, the risk-to-benefit ratio of antipsychotics is often challenged by their multiple side-effects *associated with their receptor-binding profiles and frequently linked to off-target actions beyond D2R blockade.* *^1^* *^,^* *^2^* ……

 Xanomeline, a muscarinic M1/M4-preferring receptor agonist, has demonstrated improvements in symptoms of schizophrenia in early and late-stage randomized placebo-controlled controlled trials with medium-to-large effect sizes, with potential cholinergic adverse events mitigated by its combination with trospium, a peripheral muscarinic antagonist. ^10^^ –^^13^ *In September 2024, the U.S. Food and Drug Administration approved xanomeline combined with trospium for schizophrenia, marking it as the first antipsychotic targeting muscarinic receptors.* *(1)* “

**#2.3: A systematic review and a meta-analysis are proposed for examining the impact of muscarinic agonists and allosteric modulators in animal models of psychosis. The level of complexity is high with respect to the varying types of compounds (agonists vs. partial agonists, receptor subtype selectivity, binding affinity, etc.), which will make it difficult to accurately categorize compounds. For example, positive allosteric modulators often exhibit a range of effects that include modulation of acetylcholine potency and efficacy in addition to allosteric agonist activity. The degree to which such activities have been adequately assessed will likely vary from lab to lab and such data may not be readily available in the papers that address in vivo efficacy in animal models of psychosis. These concerns are addressed to some extent in the “Exploration of heterogeneity” section, but a more explicit discussion of how concepts of potency and efficacy, for example, will be handled is warranted.**

Our reply:

 We would like to thank the reviewer for this insightful comment. We have now expanded the “Exploration of heterogeneity” section to elaborate on how these pharmacological properties will be examined, taking into account different modes of action and efforts to calculate indices that minimize potential variability and heterogeneity in measuring these properties (e.g., if possible, differences in the log((maximal efficacy/EC50)).(2) We will aim to use reliable sources for obtaining information about these properties (e.g., IUPHAR/BPS database)(3); however, we will not conduct a systematic review of studies to synthesize them, and we have noted this limitation in the “Discussion” section.

Changes in the manuscript:

  

- We expanded the methods in the “Exploration of heterogeneity” accordingly:

*We will examine the pharmacological properties of muscarinic receptor agonists, considering mode of action (e.g., orthosteric agonism, positive allosteric modulation) and other key characteristics as sources of heterogeneity, using data primarily from established databases (e.g., IUPHAR/BPS)* *^43^* *and original publications that characterize these compounds. Specifically, we will assess potency (half-maximal effective concentration, EC50) and efficacy (maximal response, distinguishing between full and partial agonism) for orthosteric agonists, and modulation of acetylcholine potency and efficacy (cooperative factors α and β), intrinsic efficacy (τ _B_), and affinity for the allosteric site (K _B_) for PAMs.* *(2,4)* *These properties will be examined across various muscarinic receptor subtypes, with a preference for human receptors while considering potential cross-species differences, and across downstream signaling pathways (e.g., via Gα _s_, Gα _q_, Gα _i/o _subunits,* *β* *-arrestin). Given the variability across assays and experimental settings, we will consider calculating a common standardized index, if possible, such as estimating differences in log((maximal response)/EC50) between the examined drugs and the natural ligand acetylcholine.* *(2)* *This approach may enable comparisons across compounds, receptor and signaling pathway selectivity, but summarizing the different properties into a single index may result in information loss, necessitating additional analyses* *.* *Moreover, the exact procedure cannot be predetermined due to anticipated heterogeneity in the data across the different compounds examined* *. Additionally, we will assess off-target actions by evaluating the mode of action and affinity of each compound toward other neurotransmitter receptors* *.”*

  

- We added to the limitations that a systematic review of in vitro assays and pharmacokinetic studies will not be conducted:

*“…* *Additionally, we will explore whether heterogeneity in behavioral effects may be explained by differences in drug interactions with muscarinic receptors. Given the limited availability and application of in vivo readouts for muscarinic agonism, we will use in vitro data. We will not conduct a systematic review of in vitro assays and pharmacokinetic studies to synthesize information about the pharmacological properties of the drugs, but instead will aim to use reliable sources and standardized methods to assess these as sources of heterogeneity (see “Exploration of heterogeneity”)…”*

**#2.4: In addition to the sources of heterogeneity discussed, the physicochemical properties of compounds may impact the effectiveness of treatments; consideration of ADME properties of compounds could provide an important perspective. For example, multiparameter optimization values could help explain variability in responses for compounds with similar receptor activity profiles.**

Our reply:

 Thank you for raising this important point. Similarly to the above, we expanded the “Exploration of heterogeneity” section to include physicochemical parameters and their integration with the Central Nervous System Multiparameter Optimization Desirability approach. (5,6)

Changes in the manuscript:

  

- We expanded accordingly the “Exploration of heterogeneity” section:

*“… We will explore potential sources of heterogeneity for …. type of muscarinic receptor agonists or PAMs, their pharmacological and physiochemical properties, and dose (details below), …*

*We* *will use the Central Nervous System Multiparameter Optimization Desirability algorithm* *(5,6)* *to examine the physicochemical properties of the drugs as source of heterogeneity. This algorithm calculates a composite desirability score based on six key properties, i.e., lipophilicity, distribution coefficient, molecular weight, topological polar surface area, number of hydrogen bond donors, and the most basic center, which aligns well with the pharmacokinetic attributes, blood-brain barrier permeability and safety* *.* *(5,6)* *Values for these properties will be estimated based on the chemical structure information using OPEn structure–activity/property Relationship App (OPERA) version 2.9.1 (a freely available, open-source provided distributed under* *MIT license* *)* *.* *(7)* *”*

**#2.5: Beyond the pharmacological properties of the compounds under investigation, the impact of muscarinic receptor activation might vary significantly depending on the different compounds utilized to induce behavioral changes (e.g., NMDA antagonists vs. dopamine agonists) or the behavioral readouts (e.g., pre-pulse inhibition of startle response vs. apomorphine induced climbing). The authors refer to some of the inherent difficulties in the “Assessment of indirectness of animal experiments” section, including concerns about the applicability of various animal models to clinical utility. In this sense, it might be helpful to examine the consistency of comparator compounds (e.g., D _2_ and 5-HT _2_ antagonists) that are used clinically in treatment of schizophrenia, and are often used as control compounds in studies of muscarinic agonists and other potential therapeutic compounds. This is perhaps alluded to in the “Data synthesis/planned comparisons” section, although additional details would be helpful.**

Our reply:

 We agree with this point, and we have added a comparison of D2R-blocking antipsychotics with inactive control conditions (e.g., vehicle) to examine the consistency of their effects in the included experiments and to inform the “Assessment of indirectness of animal experiments”. This is now described in the “Comparison groups”

Changes in the manuscript:

  

- We added this comparison in the “Comparison groups”:

*“Table 1 outlines the planned comparisons, ….*

*Additionally, we will examine the comparison between active and inactive control conditions to provide insights into the consistency of the effects of D2R-blocking antipsychotic in the included experiments and to inform the assessment of indirectness (see “Assessment of indirectness of the animal experiments”).”*

**#2.6: An additional consideration is the presence or absence of adverse effects for the compounds under investigation. The clinical utility of muscarinic agonists has been limited by adverse effect profiles, which vary from compound to compound, but likely include both off-target (e.g., M _3_receptor-mediated) and on-target (i.e., M _1_ or M _4_ receptor-mediated) effects. Adverse effects such as salivation, lacrimation, diarrhea, or convulsions would provide important information regarding the *in vivo *pharmacological profile of compounds and the separation between beneficial and undesirable effects (i.e., the therapeutic window).**

Our reply:

 Thank you for this point. We agree that examining adverse events is important for assessing the risk-to-benefit ratio of these drugs. However, we had chosen already from the start of our protocol to focus on behavioural outcomes and not include adverse events due to their sparse and inconsistent reporting in studies of animal models of psychosis (as also noted in our previous review on TAAR1 agonists)(8) and their examination in other study designs excluded from our review (e.g., using naïve animals not subjected to models of psychosis). Furthermore, cholinergic adverse events may potentially be mitigated in clinical settings through the combination of peripheral antagonists, as seen with xanomeline-trospium.(9)Nonetheless, we will aim to extract adverse event data if found in the included studies and consider secondary publications where relevant. These considerations are addressed in the “Discussion” section under limitations.

 Changes in the manuscript:

  

- We expanded the limitations in the “Discussion” section to include consideration of cholinergic adverse events, similar to the previously reported neurobiological measures:

*“We will not analyse neurobiological measures due to their heterogeneous use across studies and the lack of an established biomarker in schizophrenia,* *^6^* *..* *Similarly, we will not examine cholinergic adverse events in animals, such as salivation, lacrimation and diarrhea, which may offer insights into the risk-benefit profile but could be mitigated in clinical settings with peripheral muscarinic receptor antagonists, as shown with the xanomeline-trospium combination.* *^10^* *^–^* *^13^* We will aim to extract and potentially analyse these measures in secondary publications *.”*

**#2.7: Overall, the topic is of timely interest and worth pursuing with some additional attention to the details to be evaluated.**

Our reply:

 Thank you once again for your overall endorsement and for the thoughtful comments that have substantially strengthened our protocol. In addition to the above-mentioned changes, we have also updated the “Study status” to reflect the changes during the revision:

 “…There were no changes from the original PROSPERO registration of the protocol, except for expanding the methods with additional details *(also in response to the reviewer’s comments),* *(10,11)* *revising the search strategy to include terms for allosteric modulation as suggested by a reviewer,* *(10)* *adding physicochemical properties of the compounds in the “Exploration of Heterogeneity” per another reviewer’s comment,* *(11)* and deciding not to search Scopus and CINAHL after consulting with the information specialist (which is not expected to affect the coverage of our search)….”

**References**

 1.U.S. Food and Drug Administration. FDA Approves Drug with New Mechanism of Action for Treatment of Schizophrenia 2024 [Available from: https://www.fda.gov/news-events/press-announcements/fda-approves-drug-new-mechanism-action-treatment-schizophrenia.

 2. **Kenakin T.** A Scale of Agonism and Allosteric Modulation for Assessment of Selectivity, Bias, and Receptor Mutation. Mol Pharmacol. 2017;92(4):414-24.

 3. **Harding SD, et al.** The IUPHAR/BPS guide to PHARMACOLOGY in 2022: curating pharmacology for COVID-19, malaria and antibacterials. Nucleic Acids Res. 2022;50(D1):D1282-d94.

 4. **Kenakin T.** Know your molecule: pharmacological characterization of drug candidates to enhance efficacy and reduce late-stage attrition. Nature Reviews Drug Discovery. 2024:1-19.

 5. **Wager TT, et al.** Moving beyond rules: the development of a central nervous system multiparameter optimization (CNS MPO) approach to enable alignment of druglike properties. ACS Chem Neurosci. 2010;1(6):435-49.

 6. **Wager TT, et al.** Central Nervous System Multiparameter Optimization Desirability: Application in Drug Discovery. ACS Chem Neurosci. 2016;7(6):767-75.

 7. **Mansouri K, et al.** OPERA models for predicting physicochemical properties and environmental fate endpoints. Journal of Cheminformatics. 2018;10(1):10.

 8. **Siafis S, et al.** Trace amine-associated receptor 1 (TAAR1) agonism for psychosis: a living systematic review and meta-analysis of human and non-human data. Wellcome Open Research. 2024;9.

 9. **Paul SM, et al.** Muscarinic Acetylcholine Receptor Agonists as Novel Treatments for Schizophrenia. Am J Psychiatry. 2022;179(9):611-27.

 10. **Foster D.** Peer Review Report For: Muscarinic receptor agonists in animal models of psychosis: protocol for a systematic review and meta-analysis [version 1; peer review: 2 approved with reservations]. F1000Research. 2024;13:1017.

 11. **Messer W.** Peer Review Report For: Muscarinic receptor agonists in animal models of psychosis: protocol for a systematic review and meta-analysis [version 1; peer review: 2 approved with reservations]. F1000Research 2024;13:1017.

# Reviewer report: Reviewer response for version 1

**DOI:** 10.5256/f1000research.170522.r325666

**Published:** 2024-10-11

**Authors:** Daniel Foster

**Affiliations:** 1 Department of Pharmacology, Physiology and Neuroscience, University of South Carolina School of Medicine, Columbia, USA

The authors provide a study protocol to conduct a systematic review and meta-analyses to assess pharmacological targeting of muscarinic receptors in preclinical studies relevant to schizophrenia. This is an especially timely study that has the potential to summarize a vast preclinical literature and provide insights into the efficacy of targeting muscarinic receptors and possible sources of heterogeneity in these data sets that could arise due to drugs that have different selectivity for muscarinic receptor subtypes, or different modes of pharmacological activity. The choice of primary and secondary behaviors is well justified, and the exclusion of studies that only assess neurophysiological or histopathological measures is clearly stated.

 One potential caveat of this study protocol is the use of the pharmacological term "agonist" throughout the protocol. In specific sentences, the authors acknowledge the potential role for allosteric modulators in mediating antipsychotic-like effects. For example, in the interventions section, the authors write: "We will include any pharmacological agent acting as an agonist or positive allosteric modulator at any of the five subtypes". They then state in the Exploration of Heterogeneity section that they will consider: "pharmacological characteristics (e.g., mode of action, selectivity for muscarinic or other receptors, potency, efficacy)". These statements are much appreciated, and these meta-analyses could provide important insights into how positive allosteric modulators and agonists differ in efficacy in preclinical studies across numerous symptom domains. Given the potential importance of understanding how allosteric modulators and agonists are similar or differ in their ability to modulate different preclinical assays relating to numerous schizophrenia symptom domains, it may be worth changing the title and clarifying the importance of this pharmacological distinction more clearly. The agonist vs allosteric modulator issue is also evident in the search terms included in the extended data where only "agonist" is used and "allosteric modulator" is absent. In these searches, numerous allosteric modulators are included by name. However, this approach has the potential to miss any allosteric modulators not included by name in the search terms. The importance of studying potential discrepancies between agonist and allosteric modulators is highlighted by ongoing clinical trials for both types of compounds, with allosteric modulators currently being investigated in the clinic by AbbieVie, Neumora, and Neurosterix, and muscarinic agonist therapy having been approved for Karuna/Bristol Myers Squibb and under clinical investigation by several other companies including Neurocrine, Anavex, and Maplight.

 Another potential issue that warrants attention is the use of genetically modified knockout animals. Some of the most compelling evidence we have for determining which muscarinic receptor subtypes mediate specific behavioral effects comes from studies that combine selective pharmacology tools in mice where the receptor has been deleted either globally or from specific populations of neurons. However, the current study design does not clearly outline how or if studies in these knockout animals will be evaluated.

 The use of a similar methodology by the authors previously to assess TARR1 agonists in non-human studies is a strength, and the team assembled is well-suited to carry out this study. Overall, this study protocol is thoughtfully written and addresses important and timely questions with the potential to unveil critical insights into the ability of compounds targeting muscarinic receptors to modulate schizophrenia-relevant behaviors across numerous symptom domains in preclinical studies.

Is the study design appropriate for the research question?

Yes

Is the rationale for, and objectives of, the study clearly described?

Yes

Are sufficient details of the methods provided to allow replication by others?

Yes

Are the datasets clearly presented in a useable and accessible format?

Not applicable

Reviewer Expertise:

I study muscarinic receptor modulation of preclinical physiology and behaviors relevant to schizophrenia.

I confirm that I have read this submission and believe that I have an appropriate level of expertise to confirm that it is of an acceptable scientific standard, however I have significant reservations, as outlined above.

# Response

**Published:** 2024-12-20

**Authors:** Spyridon Siafis

**Affiliations:** Psychiatry and Psychotherapy, Technische Universitat Munchen, Munich, Bavaria, Germany

**#1.1: The authors provide a study protocol to conduct a systematic review and meta-analyses to assess pharmacological targeting of muscarinic receptors in preclinical studies relevant to schizophrenia. This is an especially timely study that has the potential to summarize a vast preclinical literature and provide insights into the efficacy of targeting muscarinic receptors and possible sources of heterogeneity in these data sets that could arise due to drugs that have different selectivity for muscarinic receptor subtypes, or different modes of pharmacological activity. The choice of primary and secondary behaviors is well justified, and the exclusion of studies that only assess neurophysiological or histopathological measures is clearly stated.**

Our reply:

 We would like to thank the reviewer for recognizing the relevance of our planned work, for the overall endorsement of our methods, as well as for the constructive comments that further improved our methodology.

 Please find our point-by-point responses to the comments below.

**#1.2 One potential caveat of this study protocol is the use of the pharmacological term "agonist" throughout the protocol. In specific sentences, the authors acknowledge the potential role for allosteric modulators in mediating antipsychotic-like effects. For example, in the interventions section, the authors write: "We will include any pharmacological agent acting as an agonist or positive allosteric modulator at any of the five subtypes". They then state in the Exploration of Heterogeneity section that they will consider: "pharmacological characteristics (e.g., mode of action, selectivity for muscarinic or other receptors, potency, efficacy)". These statements are much appreciated, and these meta-analyses could provide important insights into how positive allosteric modulators and agonists differ in efficacy in preclinical studies across numerous symptom domains. Given the potential importance of understanding how allosteric modulators and agonists are similar or differ in their ability to modulate different preclinical assays relating to numerous schizophrenia symptom domains, it may be worth changing the title and clarifying the importance of this pharmacological distinction more clearly.**

Our reply:

 We would like to thank the reviewer for raising this important point. We had already planned to examine both orthosteric receptor agonists and positive allosteric modulators, as well as their differences, which we had highlighted in several parts of the manuscript. However, for simplicity, we had generally used the term 'agonism' throughout the protocol. We agree with the reviewer on the importance of this distinction, and we have clarified this in response to the reviewer’s comments.

Changes in the manuscript:

  

- We have revised the title to include 'positive allosteric modulation': 

*“*Muscarinic receptor agonists *and positive allosteric modulators* in animal models of psychosis: protocol for a systematic review and meta-analysis *.”*

  

- We have incorporated 'positive allosteric modulation' more frequently throughout the manuscript to emphasize this distinction.

 

- We have clarified its significance further in the introduction:

“…., and the potential differences between orthosteric agonists and PAMs. *For example, PAMs modulate receptor activity by binding to a site distinct from the natural ligand, offering theoretical advantages such as increased selectivity and safety over orthosteric agonists.* *(1,2)*”.

  

- In relationship to the comments of another reviewer,(3) we have also expanded the “Exploration of heterogeneity” section by providing more details in the pharmacological properties considered for orthosteric agonists and positive allosteric modulators:

*“* *We will examine the pharmacological properties of muscarinic receptor agonists, considering mode of action (e.g., orthosteric agonism, positive allosteric modulation) and other key characteristics as sources of heterogeneity, using data primarily from established databases (e.g., IUPHAR/BPS)* *^43^* *and original publications that characterize these compounds. Specifically, we will assess potency (half-maximal effective concentration, EC50) and efficacy (maximal response, distinguishing between full and partial agonism) for orthosteric agonists, and modulation of acetylcholine potency and efficacy (cooperative factors α and β), intrinsic efficacy (τ _B_), and affinity for the allosteric site (K _B_) for PAMs.* *(1,4)* *These properties will be examined across various muscarinic receptor subtypes, with a preference for human receptors while considering potential cross-species differences, and across downstream signaling pathways (e.g., via Gα _s_, Gα _q_, Gα _i/o _subunits,* *β* *-arrestin). Given the variability across assays and experimental settings, we will consider calculating a common standardized index, if possible, such as estimating differences in log((maximal response)/EC50) between the examined drugs and the natural ligand acetylcholine.* *(4)* *This approach may enable comparisons across compounds, receptor and signaling pathway selectivity, but summarizing the different properties into a single index may result in information loss, necessitating additional analyses. Moreover, the exact procedure cannot be predetermined due to anticipated heterogeneity in the data across the different compounds examined.”*

**#1.3 The agonist vs allosteric modulator issue is also evident in the search terms included in the extended data where only "agonist" is used and "allosteric modulator" is absent. In these searches, numerous allosteric modulators are included by name. However, this approach has the potential to miss any allosteric modulators not included by name in the search terms. The importance of studying potential discrepancies between agonist and allosteric modulators is highlighted by ongoing clinical trials for both types of compounds, with allosteric modulators currently being investigated in the clinic by AbbieVie, Neumora, and Neurosterix, and muscarinic agonist therapy having been approved for Karuna/Bristol Myers Squibb and under clinical investigation by several other companies including Neurocrine, Anavex, and Maplight.**

Our reply:

 We would like to thank the reviewer for highlighting this potential caveat in our search strategy. In collaboration with Dr. Farhad Shokraneh, an experienced information specialist (see Acknowledgments in the manuscript), we have revised the search strategy across multiple electronic databases to include terms relevant to allosteric modulation, ensuring we capture any studies on this category of medications. As it was already noted in the methods section, we will also supplement our searches using the psychosis-SOLES,(5)a dedicated database of preclinical psychosis studies, allowing us to conduct broader searches without restricting terms to “agonism” or “allosteric modulation”. These methods will enhance our search coverage and reduce the risk of missing relevant studies on positive allosteric modulators.

Changes in the manuscript:

  

- We have revised the methods to clarify that the search strategies will encompass both agonism and allosteric modulation:

'For muscarinic agents, we will use broad terms like muscarinic agonists *or allosteric modulators* and…'

  

- Additionally, we have updated the draft search strategy in MEDLINE via Ovid within the extended data to include terms for “allosteric modulation”. See the revised extended data( https://doi.org/10.5281/zenodo.14534308):

*“...* *﻿* *#3. Muscarinic Agonists/ or Muscarine/ or (Muscarin or Muscarine or Muscarinic or M1 or M2*

or M3 or M4 or M5 or Muscarinomimetic*).ti,ab,tw.

#4. Allosteric Regulation/ or Allosteric Site/ or (Alloster* or PAM or PAMs or Modulat* or

Agonis*).ti,ab,tw.

*#5. 3 and 4….*”

**#1.4: Another potential issue that warrants attention is the use of genetically modified knockout animals. Some of the most compelling evidence we have for determining which muscarinic receptor subtypes mediate specific behavioral effects comes from studies that combine selective pharmacology tools in mice where the receptor has been deleted either globally or from specific populations of neurons. However, the current study design does not clearly outline how or if studies in these knockout animals will be evaluated.**

Our reply:

 Thank you for this point. We agree that genetically modified knockout animals can be important tools for delineating the potential receptor mechanisms of pharmacological interventions. We had already planned to compare muscarinic receptor agonists or PAMs with inactive control conditions in the context of genetic or pharmacological muscarinic receptor antagonism to determine if any observed differences result from muscarinic receptor activation, as it had been outlined in Table 1 and “Animal Population and Model Induction”. However, we agree that this was not clearly described, and we have therefore revised the methods accordingly.

Changes in the manuscript:

  

- We revised the “Animal Population and Model Induction” section to better describe the inclusion of animal cohorts with genetic knockouts of muscarinic receptors or those receiving pharmacological muscarinic antagonists:

“….Moreover, in the eligible studies, we will extract data from “naïve” animal cohorts (i.e., animals that have not undergone models of psychosis), and animal cohorts that have undergone both models of psychosis and muscarinic receptor antagonism via genetic *(i.e,* *genetically modified animals with knockout of all or specific muscarinic receptors, either globally or in specific neuron populations)* *(6)* or pharmacological manipulation *(e.g.,* *co-administration of selective or non-selective muscarinic receptor antagonists like scopolamine)* *(7)* *(see further details in “Comparison groups”)….”*

  

- We expanded the “Comparison groups” to mention explicitly the comparison of muscarinic drugs versus inactive control conditions (e.g., vehicle) in animal models of psychosis in the context of muscarinic receptor antagonism:

“… *We will also compare muscarinic receptor agonists or PAMs with inactive control conditions in contexts of genetic or pharmacological muscarinic receptor antagonism (as described in “Animal population and model induction”) to determine if any observed differences result from muscarinic receptor activation. Separate analyses may be conducted for the antagonism of specific muscarinic receptor subtypes and/or within specific neuronal populations, if sufficient data are available..”*

**#1.5: The use of a similar methodology by the authors previously to assess TARR1 agonists in non-human studies is a strength, and the team assembled is well-suited to carry out this study. Overall, this study protocol is thoughtfully written and addresses important and timely questions with the potential to unveil critical insights into the ability of compounds targeting muscarinic receptors to modulate schizophrenia-relevant behaviors across numerous symptom domains in preclinical studies.**

Our reply:

 Thank you once again for your overall endorsement and for the thoughtful comments that have substantially strengthened our protocol. In addition to the above-mentioned changes, we have also updated the “Study status” to reflect the changes during the revision:

 “…There were no changes from the original PROSPERO registration of the protocol, except for expanding the methods with additional details *(also in response to the reviewer’s comments),* *(3,8)* *revising the search strategy to include terms for allosteric modulation as suggested by a reviewer,* *(8)* *adding physicochemical properties of the compounds in the “Exploration of Heterogeneity” per another reviewer’s comment,* *(3)* and deciding not to search Scopus and CINAHL after consulting with the information specialist (which is not expected to affect the coverage of our search)….”

**References**

 1. **Kenakin T.** Know your molecule: pharmacological characterization of drug candidates to enhance efficacy and reduce late-stage attrition. Nature Reviews Drug Discovery. 2024:1-19.

 2. **Foster DJ, et al.** Allosteric Modulation of GPCRs: New Insights and Potential Utility for Treatment of Schizophrenia and Other CNS Disorders. Neuron. 2017;94(3):431-46.

 3. **Messer W.** Peer Review Report For: Muscarinic receptor agonists in animal models of psychosis: protocol for a systematic review and meta-analysis [version 1; peer review: 2 approved with reservations]. F1000Research 2024;13:1017.

 4. **Kenakin T.** A Scale of Agonism and Allosteric Modulation for Assessment of Selectivity, Bias, and Receptor Mutation. Mol Pharmacol. 2017;92(4):414-24.

 5. **Smith S, et al**. Psychosis-SOLES 2024 [Available from: https://camarades.shinyapps.io/psychosis-solesMarch 4, 2024.

 6. **Thomsen M, et al.** Physiological roles of CNS muscarinic receptors gained from knockout mice. Neuropharmacology. 2018;136(Pt C):411-20.

 7. **Klinkenberg I, et al.** The validity of scopolamine as a pharmacological model for cognitive impairment: a review of animal behavioral studies. Neurosci Biobehav Rev. 2010;34(8):1307-50.

 8. **Foster D.** Peer Review Report For: Muscarinic receptor agonists in animal models of psychosis: protocol for a systematic review and meta-analysis [version 1; peer review: 2 approved with reservations]. F1000Research. 2024;13:1017.
